# Dynamic Behavior of the Stenting & Shielding Hernia System Fosters Neomyogenesis in Experimental Porcine Model

**DOI:** 10.3390/bioengineering12080883

**Published:** 2025-08-19

**Authors:** Giuseppe Amato, Roberto Puleio, Antonino Agrusa, Vito Rodolico, Luca Cicero, Giovanni Cassata, Giuseppe Di Buono, Emanuele Battaglia, Claudia Neto, Giorgio Romano, William Ra, Giorgio Romano

**Affiliations:** 1 Department of Precision Medicine in Medical, Surgical and Critical Areas, University of Palermo, 90127 Palermo, Italy; antonino.agrusa@unipa.it (A.A.); giuseppe.dibuono@unipa.it (G.D.B.); emanuele.battaglia@policlinico.pa.it (E.B.);; 2 Department of Pathologic Anatomy and Histology, Istituto Zooprofilattico Sperimentale della Sicilia (IZSS Palermo), 90129 Palermo, Italy; roberto.puleio@izssicilia.it; 3 Department PROMISE, Section Pathological Anatomy, University of Palermo, 90127 Palermo, Italy; vito.rodolico@unipa.it; 4 CEMERIT—Experimental Zooprophylactic Institute of Sicily Palermo, 90129 Palermo, Italy; luca.cicero@izssicilia.it (L.C.); giovanni.cassata@izssicilia.it (G.C.); 5 Postgraduate School of General Surgery, University of Palermo, 90127 Palermo, Italy; neto.claudia98@gmail.com (C.N.); giorgioromano95@gmail.com (G.R.); williamra95@gmail.com (W.R.)

**Keywords:** hernia, regenerative scaffolds, tissue regeneration, neo-myogenesis, experimental research

## Abstract

Despite significant advancements, prosthetic hernia repair continues to face unacceptably high complication rates. These likely stem from poor biological responses, such as stiff scar tissue leading to mesh shrinkage. To overcome these issues, the Stenting and Shielding (S&S) Hernia System, a newly designed 3D dynamic device, has been developed for dissection-free laparoscopic placement to permanently obliterate hernia defects. Unlike conventional meshes, this device induces a regenerative biological response, promoting viable tissue growth rather than fibrotic plaque formation. In a porcine experimental model, the S&S device demonstrated the development of a great amount of muscle fibers, alongside nervous and vascular structures, within well-perfused connective tissue. Histological analysis of biopsy specimens excised from the experimental animals revealed progressive muscle fiber maturation from early myocyte development in the short term to fully developed muscle bundles in the long term. The enhanced biological response observed with the S&S device suggests a promising shift in hernia repair, potentially reversing the degenerative processes of hernia formation and promoting tissue regeneration. The S&S Hernia System described here can be classified not merely as a conventional hernia implant, but as part of a new category of hernia devices: the dynamic regenerative scaffold.

## 1. Introduction

The biological response to hernia prosthetics remains a topic of ongoing debate among herniologists. One key issue is mesh shrinkage, caused by the retraction of fibrotic tissue ingrown into the prosthetic material [1,2,3,4]. This phenomenon is often cited as a contributing factor to the reduction in mesh surface area, potentially leading to inadequate coverage of the hernia defect and an increased risk of recurrence [5,6,7]. Another critical concern is the stiff fibrotic incorporation that occurs shortly after implantation. This characteristic of conventional hernia meshes is believed to be a primary cause of the discomfort and chronic pain frequently reported by patients following prosthetic abdominal hernia repair [2,4,8,9,10]. The development of a rigid, inelastic fibrotic plaque impairs the natural mobility of the abdominal wall, sometimes resulting in painful restrictions of muscular movements.

The literature suggests that this issue may be linked to the static nature of flat meshes, which, when encased by immobile and rigid tissue, can hinder normal muscle function in the repaired groin [11]. Additionally, recent studies on hernia genesis, particularly focused on inguinal protrusions, have highlighted the degenerative origins of the disease [12]. This renewed interest in developing more physiological and pathogenetically aligned approaches to abdominal wall hernia repair has led to the creation of a new category of hernia repair devices based on dynamic 3D scaffolds with regenerative features [13,14,15]. The first device of this category, known as ProFlor, was introduced by our group as a self-expanding, dynamic regenerative scaffold made of polypropylene, specifically designed for inguinal hernia repair. ProFlor’s configuration was validated in extensive porcine studies and subsequently applied in human surgery to achieve defect obliteration while avoiding the need for fixation. Concerning the biological response, thanks to its proprietary dynamic responsivity, ProFlor demonstrated the capacity to induce neomyogenesis, neonervegenesis, and neoangiogenesis. These results have been documented in multiple peer-reviewed publications and formed the basis for further innovations [16,17,18]. Building on the clinical and experimental results of ProFlor, the development of the second-generation Stenting and Shielding Hernia System was initiated. This device retains the principles of dynamic defect stenting but incorporates an additional shielding component to provide mechanical protection against intra-abdominal pressure. In contrast to ProFlor, it can be positioned intra-abdominally in seconds without tissue trauma or dissection. The evolution from ProFlor to S&S represents a continuous, evidence-based research pathway in the field of 3D dynamic regenerative hernia scaffolds, aligning with recent advancements in bioengineered prosthetics as described in the latest literature [19]. Building on this innovative line of research, the Stenting and Shielding (S&S) Hernia System—a dynamic, responsive device—has been developed. Constructed from TPE material, these devices consist of a rayed element assembled around a central mast connected to an oval shield. Delivered laparoscopically, they expand within the hernia defect to achieve permanent obliteration. Their compliance with the natural movements of the abdominal wall, combined with their ability to elicit a regenerative biological response, sets them apart from traditional flat and static meshes. The dynamic responsivity of the S&S device likely promotes the incorporation of viable tissue, including newly developed arteries, veins, nerves, and notably, myocytes resembling the typical components of the abdominal wall. The present study aims to demonstrate the development and maturation of muscle elements within the S&S Hernia System at various postoperative stages, using a cohort of experimental pigs that underwent repair of induced abdominal wall defects with the S&S Hernia System.

## 2. Materials and Methods

The experimental trial was conducted in accordance with the Animal Care Protocol for Experimental Surgery, as stipulated by the Italian Ministry of Health. The protocol was officially approved on 1 June 2021 with the Decree No. 379/2021-PR.

Between February 2022 and November 2024, ten female pigs, each with bilateral muscular defects previously purchased in the lower abdomen, were selected for the trial. Each animal underwent laparoscopic placement of two Stenting and Shielding (S&S) Hernia Devices. The S&S device is specifically engineered to provide a dissection-free, minimally invasive repair for a range of abdominal wall hernias, including inguinal, incisional, femoral, Spigelian, and obturator hernias. The pigs, aged 4 to 6 months and weighing between 40 and 60 kg, were chosen for this study. All laparoscopic implantations were carried out under general anesthesia. The anesthesia protocol included premedication with zolazepam and tiletamine (6.3 mg/kg) and xylazine (2.3 mg/kg), induction with propofol (0.5 mg/kg), and maintenance with isoflurane in combination with pancuronium (0.07 mg/kg). Postoperative care involved antibiotic prophylaxis with oxytetracycline (20 mg/kg/day) for three days.

### 2.1. Stenting and Shielding Hernia System: The Structure

The S&S Hernia System used in this study is constructed from a medical-grade polypropylene-based Thermoplastic Elastomer (TPE), identified as Thermolast^®^ M type TM9HET, manufactured by KRAIBURG TPE GmbH & Co. KG (Waldkraiburg, Germany). This material, employed in the injection molding of the device, has its technical specifications detailed in Table 1.

**Figure 1:**
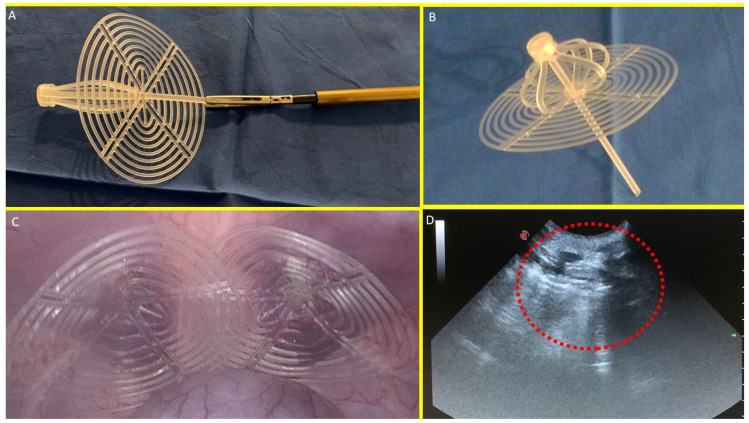
(**A**) The Stenting and Shielding (S&S) Hernia System in its pre-implantation state, ready for insertion into the abdominal cavity of the experimental pig. (**B**) The S&S device in its deployed configuration. (**C**) The two shields of the S&S Hernia System laparoscopically positioned in the lower abdominal wall, facing the abdominal viscera. (**D**) Ultrasound scan of the S&S device three months post-surgery, showing the 3D scaffold occupied by newly ingrown tissue (red circle).

The S&S device consists of two primary components: the first is an eight-rayed structure arranged as a cylindrical compound sized 48 mm long and 8 mm wide and assembled around a central mast; the second component has a 3D outline of an oval shield dimensioned at 10 × 8 cm. The oval shield has a central ring intended to be connected with the mast. The mast has a button-like arrangement at its distal end, along with two conical enlargements (stops) near the button. Initially, the device is configured as a compact cylindrical unit, with the oval shield threaded onto the mast via its central ring (Figure 1A,B).

This design allows the device to be delivered into the abdomen of the pig through a 12 mm trocar channel. Once positioned at the hernia defect, a metallic tube is used to push the oval shield, advancing the device into the pre-existing muscular defect. When the cylindrical structure is inside the defect, the shield is pressed beyond the first or the second conic enlargements on the mast that practically act as stops: this allows the cylindrical structure to modify its shape into a 3D scaffold that permanently occupies the hernia opening. This final configuration blocks the shield preventing any backward movement and firmly fastening the 3D scaffold within the defect. In this configuration, the shield covers and overlaps the muscular defect remaining in direct contact with the abdominal content (Figure 1C). The final diameter of the 3D scaffold utilized for the experiment is approximately 4.5 cm.

### 2.2. Follow-Up Protocol

The follow-up protocol involved the sacrifice of 2 pigs between 4 and 6 weeks postoperatively (short-term period), 3 between 3 and 4 months (mid-term) and 5 between 5 and 8 months (long-term). This unequal distribution was due to both budgetary constraints and ethical considerations associated with long-term animal housing. The extended observation in the long-term group aimed to maximize data acquisition at the most biologically mature stage of the regenerative process. Minor variation in sacrifice timing within each group resulted from logistical factors, including surgical suite availability at the animal facility.

Scheduled ultrasound (Figure 1D) and laparoscopic controls were carried out at defined postoperative intervals to ensure the 3D scaffold remained properly positioned and to visually determine if adhesions between abdominal viscera and the shield occurred.

**Figure 2:**
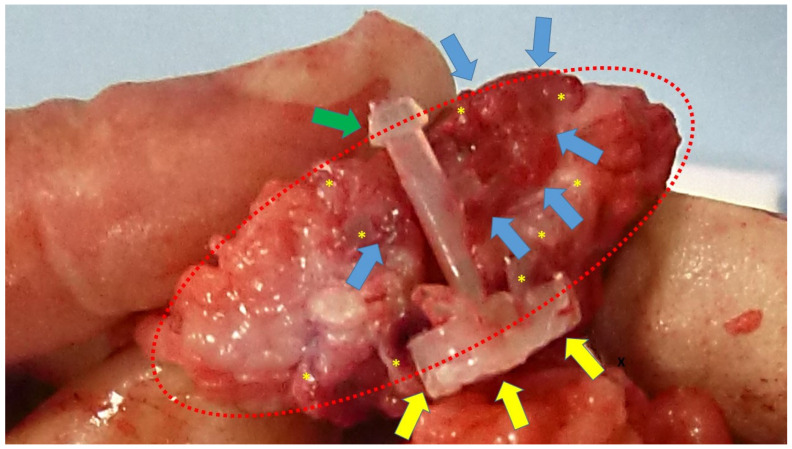
Scaffold of a bisected S&S device (red circle) removed six months post-surgery. The 3D chamber formed by the bent rays of the device (*) is clearly filled with newly developed viable muscle structures (blue arrows). The image also highlights the distal portion of the mast with the conic stop (green arrow) and the button-like enlargement on the opposite side (yellow arrows).

At the time of animal sacrifice, the S&S devices were removed in one piece through a lower midline abdominal incision. Then, the host’s native tissue around the devices was carefully excised from the device that was subsequently bisected to allow for macroscopic evaluation of the tissue ingrown into the 3D scaffold (Figure 2).

The explanted S&S devices were subsequently sent to a pathologist for detailed histological examination.

### 2.3. Histology and Histochemical Methods

The tissue specimens excised from the core of the 3D scaffold of the S&S device were fixed in 10% phosphate-buffered formalin for a minimum of 12 h before being embedded in paraffin. Sections were cut to a thickness of 4 μm and stored at room temperature until analysis. Basic histology using hematoxylin and eosin (H&E) staining was performed to evaluate the histomorphological characteristics. Sections were also analyzed using Azan–Mallory staining, which employs two acid dyes: azocarmine and aniline blue. This staining technique allows for the differentiation of various tissue components, staining collagen blue, muscle tissue reddish, and chromatin and erythrocytes red.

### 2.4. Immunohistochemistry

Further sections were assessed by immunohistochemistry to demonstrate at each temporal stage the presence of neomyogenetic activity by evidencing the density of specific clusters using anti-NGF rabbit monoclonal antibody (Abcam, Cambridge, UK, code: ab52918). After dewaxing the sections, antigen retrieval was carried out using a Tris-EDTA solution (pH 9.0), which was heated to 96 °C for 20 min. Endogenous peroxidase activity was suppressed by incubating the slides in a 3% hydrogen peroxide solution in methanol for 30 min. To minimize background staining, a 15 min treatment with Background Sniper was performed. The tissue sections were then incubated at room temperature for 1 h with primary antibodies against NGF, dilution: 1:100. As a note, the specificity of the anti-NGF antibody (Abcam, ab52918) for porcine tissues was confirmed by immunohistochemistry showing selective staining of nervous and muscular structures, as well as by manufacturer-provided Western blot data demonstrating a single band at ~32 kDa in porcine hippocampal tissue lysate, consistent with the expected molecular weight of NGF. The microphotograph provided as Supplemental Material confirms this aspect (Figure S1).

### 2.5. Histopathological Assessment

Two pathologists evaluated the histologic sections in a blinded manner respect to timing of device placement. Samples were examined under high-power light microscopy to observe the device/tissue interface and perform a semi-quantitative histological assessment. The analysis focused on assessing the width of the muscle surface as revealed by Azan–Mallory staining. The morphological features of myocytes were acknowledged and measured using digital images of stained sections. Measurements of the muscle area were taken from four non-overlapping fields at 50× magnification (total field area: 2.263493 µm^2^). Images were acquired using a bright-light microscope, equipped with a digital camera, and analyzed using image capture software (Leica DMLB microscope (Leica Microsystems, New South Wales, Australia), Nikon DS-Fi-1 digital camera (Nikon, Tokyo, Japan), NIS Basic Research Nikon software, https://downloads.microscope.healthcare.nikon.com/phase7/literature/Brochures/NIS-Elements_2CE-MPCJ-6-A.pdf, accessed on 21 May 2025).

### 2.6. Statistical Analysis

Muscle area was evaluated in 20 biopsies excised from the 3D scaffold of the S&S device excised at different times post-implantation: 4 samples 3–5 weeks postop, 6 samples 3–4 months postop, and 10 samples 5–8 months postop. A one-way ANOVA between 20 biopsies was conducted to compare the muscular ingrowth in the 3D scaffold of the S&S Hernia System with respect to time in the short, mid, and long term. Moreover, the Tukey–Kramer multiple comparison test was used to analyze significance of differences between stages. The criterion *p* < 0.05 was chosen to assign statistical significance. Data are the average of muscle area ± standard deviation in biopsies excised at different times post-implantation. SAS software (version 9.3. SAS) was used for the analyses.

## 3. Results

**Figure 3:**
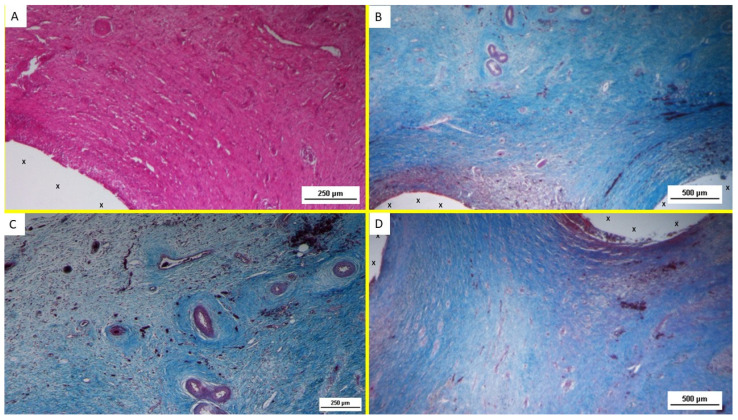
(**A**) Biopsy sample from the 3D scaffold of the S&S device excised 4 weeks post-implantation (short-term): Microphotograph showing myocytes in the early stages of development surrounded by well-perfused connective tissue and numerous vascular structures in early development, adjacent to the S&S device fabric (X). HE 50X. (**B**) Biopsy sample excised 5 weeks postop: in a context of unstructured connective tissue, multiple spots of muscular elements and several strips of muscle bundles in an initial phase of development are highlighted, particularly close to the S&S structure (X) some clusters of vascular elements in the early developmental stage are also visible. AM 25X. (**C**) Magnified image of Figure 3B highlighting details of the vascular structures in the initial stage of development. AM 50X. (**D**) Biopsy specimen excised 4 weeks postop: In a context of unstructured connective tissue, multiple spots of muscular elements and several strips of muscle bundles are highlighted, particularly in the peri-prosthetic area, in an initial phase of development.

**Figure 4:**
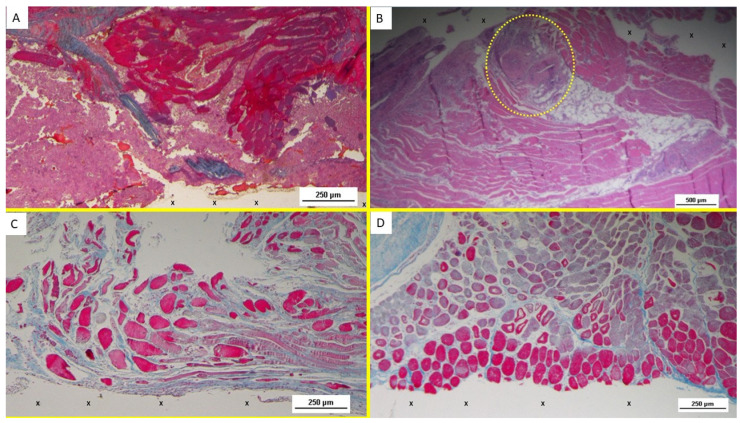
(**A**) Biopsy specimen excised 5 weeks postop: in a surround of well-organized connective tissue, numerous clusters of muscle elements, together with large areas of structured muscle fascicles, are evident adjacent to the 3D scaffold of the device (X). AM 50X. (**B**) Biopsy sample excised 3 months postoperation revealing a significant amount of muscle structures in advanced development, arranged in bundles (red elongated structures) near a large vascular element (yellow circle), in close proximity to the S&S fabric (X). HE 25X. (**C**) Biopsy specimen excised 3 months post-surgery: microphotograph showing several bundles of muscular elements (red spots) in advanced stages of development near the S&S scaffold fabric (X), within a matrix of loose and viable connective tissue (stained in blue). AM 50X. (**D**) Biopsy excised from the 3D scaffold of the S&S Hernia System 3 months post-surgery: numerous muscle structures grouped in bundles stained in red and near the 3D scaffold of the S&S fabric (X). The muscle elements close to the fabric show advanced development, while those farther away appear less mature. 50X.

At animal euthanasia, none of the pigs exhibited adhesions to the abdominal viscera. In the biopsies taken from the 3D implant excised 4–6 weeks after placement (short term), the presence of lax and well-perfused connective tissue was observed. Already in this phase, multiple spots of muscular elements and several strips of muscle bundles in progressive maturation could be detected in the unstructured connective tissue and close to the prosthetic tissue (Figure 3A–D and Figure 4A).

Overall, the typical features of the early phase of muscle development, characterized by vesiculated nuclei, prominent nucleoli, and moderate basophilia, were markedly evidenced in the newly ingrown tissue.

The development of muscle elements in biopsy samples taken from the 3D scaffold of the S&S device 3 to 4 months after placement (mid-term) showed a noticeable increase in both quantity and quality. At this stage, a greater number of muscle element clusters, along with extensive areas of muscle bundles, were observed adjacent to the device fabric and interspersed within well-organized connective tissue (Figure 4B–D).

**Figure 5:**
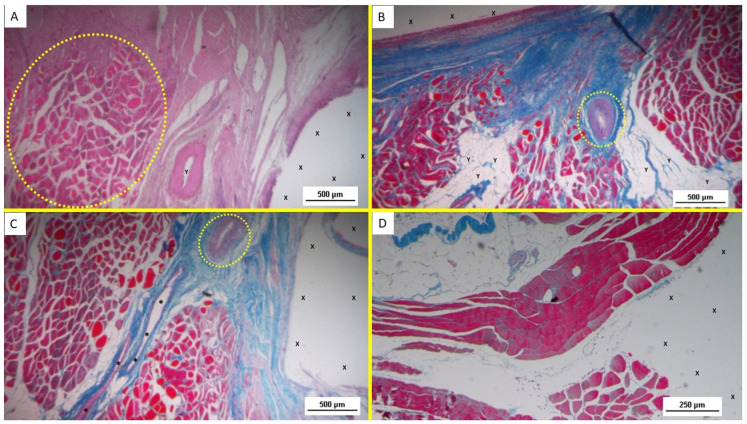
(**A**) Biopsy sample excised from the 3D scaffold of the S&S device 6 months post-implantation: microphotograph showing a large area of mature muscle elements grouped in bundles (red spots within the yellow circle) near a large arterial structure (Y) and adjacent to the S&S scaffold fabric (X). HE 25X. (**B**) Biopsy specimen excised from the 3D scaffold of the S&S device 6 months post-surgery: Microphotograph showing three clusters of fully developed muscle bundles (stained in red) interspersed between two areas of adipocytes (Y) and close to a large arterial structure (yellow circle). The blue-stained tissue adjacent to the device fabric corresponds to well-perfused connective tissue. AM 25X. (**C**) Biopsy sample excised from the 3D scaffold of the S&S device 6 months post-implantation. The image shows the S&S scaffold fabric (X) surrounded by well-hydrated, viable connective tissue (stained in blue), close to a large arterial structure (yellow circle) and two elongated veins (*) between two large areas of muscle bundles in advanced development (stained in red). AM 25X. (**D**) Tissue specimen taken from the 3D scaffold of the S&S device 7 months postoperation, highlighting elongated bundles composed of mature muscle elements (colored in red) near the S&S scaffold fabric (X). The blue convoluted structure in the upper left margin corresponds to a nerve AM 50X.

In the long-term period, 5 to 8 months post-surgery, the muscular presence within the structure of the S&S device further improved, assuming the outline of mature muscular elements grouped in the characteristic bundles typical of normal muscle structure. These muscular components were disseminated within a network of interlaced connective tissue ordered along lines of force, and accompanied by numerous mature arteries and veins (Figure 5A–D).

Highly magnified images also highlighted the typical spindle-formed shape of the myocytes, with striated elements, hyperchromatic small nuclei, and eosinophilic cytoplasm. This structure closely resembles that of normal human muscle bundles.

**Figure 6:**
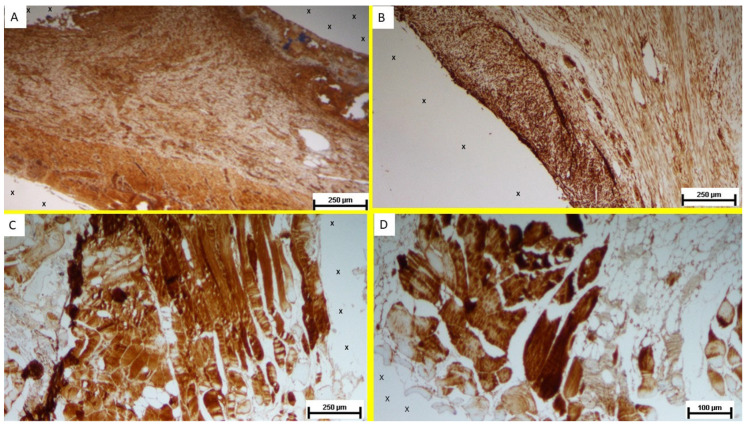
(**A**) Biopsy specimen excised 4 weeks post-implantation. Plenty of NGF-positive elements (stained in brown) close to the TPE fabric of the 3D scaffold (X). NGF 50X. (**B**) Tissue specimen excised 4 months post-implantation. Numerous NGF-positive elements (stained in brown) evidenced close to the TPE structure of the 3D scaffold (X). NGF 50X. (**C**) Biopsy specimen excised 8 months post-implantation. Several elongated bundles of NGF-positive structures (brown staining) visible near the fabric of the 3D scaffold (X). NGF 50X. (**D**) Biopsy specimen excised 8 months post-implantation. Plenty of NGF-positive elements (stained in brown) are visible near the structure of the 3D scaffold (X). NGF 100X.

Regarding the presence of myogenic growth factors, immunohistochemical analysis of biopsy samples from the S&S 3D scaffold, excised during the short-term postoperative phase, revealed a substantial presence of NGF-immunoreactive elements within the TPE matrix of the scaffold (Figure 6A). In the mid-term phase, an increase in NGF-positive cellular elements was observed within the scaffold’s fabric, coinciding with a more extensive and spatially organized development of muscle tissue (Figure 6B). The muscle bundles appeared in a stage of progressive development, with myocytes displaying spindle-formed contours, small nuclei, and eosinophilic cytoplasm. These features are characteristic of the evolving phase of muscle structure formation.

In the long-term stage, NGF-positive cells appeared in greater number and were frequently arranged in elongated, fiber-like structures within the scaffold. This persistent NGF expression supports the notion of sustained myogenic stimulation and ongoing muscle tissue regeneration in the scaffold environment (Figure 6C,D).

### Statistical Analysis

**Figure 7:**
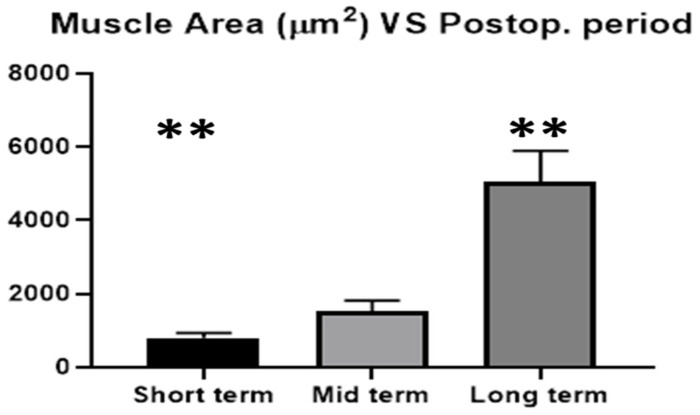
Muscular ingrowth at different periods post-surgery (short term, mid-term, and long term). Error bars indicate SD (** = *p* < 0.001).

The average area of muscle tissue of 20 biopsies excised from the 3D scaffold of the S&S device at different stages post-implantation were compared with one-way Anova and post hoc Tukey test. The results showed statistically significant differences between stages (Figure 7) and, in particular, suggested that muscular ingrowth in 3D scaffold of the S&S Hernia System progressively increases over time.

Specifically, our results indicate that, over the long term, the average muscle area is significantly greater compared to the short-term and mid-term measurements (*p* < 0.001). However, there is no statistical difference between short-term and mid-term values (*p* > 0.05).

## 4. Discussion

Prosthetic implants are currently regarded as the most effective tools for abdominal hernia repair [25]. The introduction of biologically compatible flat meshes in hernia treatment has significantly improved outcomes [26]. However, despite the wide range of available repair techniques and implant types, no definitive gold standard has been established. Nevertheless, notwithstanding notable advancements, recent concerns have emerged within the scientific community regarding the high incidence of complications specifically associated with conventional static implants [2,27].

A substantial body of research addresses the biological response to these traditional implants [28,29,30,31]. The term “scar plate” commonly refers to the rigid, fibrotic plaque formed by conventional flat meshes. These meshes are dynamically passive and unable to accommodate abdominal movements. During motion, the rigid, uneven surface of the fibrotic, motionless implants can cause friction against the surrounding muscular structure, leading to discomfort and pain [2,4,8]. This issue appears to be linked to the suboptimal tissue development within the implants, where the formation of poor quality scar tissue results in the well-documented phenomenon of mesh shrinkage. Shrinkage of the implant, leading to a reduction in surface area (up to 30%), has been identified as a significant factor contributing to hernia recurrence [5,6]. Furthermore, mesh fixation presents another challenge, as it restricts the natural movement of the abdominal wall and can lead to complications such as tissue tears, bleeding, and hematoma [1,2,4,7]. These issues underscore the limitations of current approaches and highlight the need for new solutions in hernia treatment. The scientific community is increasingly focused on exploring new strategies that move beyond the traditional concept of reinforcing the abdominal wall through the incorporation of fibrotic tissue in mesh implants.

Building on the latest findings in the pathophysiology of herniated abdominal walls, particularly with regard to inguinal hernias, a new perspective has recently gained traction. A series of scientific studies have demonstrated that the degenerative damage observed in herniated groins typically presents the histological characteristics of chronic compressive injury [12]. Notably, there is no other significant source of chronic compression in this region aside from the constant pressure exerted by the abdominal viscera against the abdominal wall. Consequently, this steady visceral impact may be recognized as a key factor in the development of hernia disease.

This evidence strongly suggests that an effective approach to hernia repair should prioritize the regeneration of degenerated abdominal wall components, a strategy that markedly differs from the traditional method of reinforcing the abdominal wall with a fibrotic plaque, typical of conventional meshes. This noteworthy paradigm shift has catalyzed the development of a new device for dissection-free laparoscopic hernia repair: the Stenting and Shielding (S&S) Hernia System.

The S&S device, constructed from a polypropylene-based Thermoplastic Elastomer (TPE), features a cylindrical, multi-rayed structure connected to an oval shield made from the same material. Once laparoscopically inserted into the abdomen, the device is placed within the hernia defect, where a proprietary mechanism transforms the cylindrical structure into a 3D scaffold that fully occupies the defect. The shield then covers and overlaps the hernia opening, with its posterior surface facing the parietal peritoneum. The S&S device, which requires no fixation, remains compliant with the natural movements of the abdominal wall, providing permanent obliteration of the defect. In experimental porcine models, this innovative device demonstrated a regenerative biological response, characterized by the ingrowth of newly developed tissue resembling the typical components of the abdominal wall, including arteries, veins, nerves, and notably, newly formed muscle structures.

The dynamic and responsive nature of the S&S device stands in stark contrast to the static behavior of conventional flat meshes, suggesting that the difference in biological response is directly tied to the distinct physical properties of the implants. A static, regressive biological response is observed in traditional meshes, while a regenerative biological response is evident in the S&S device.

From a pathogenetic viewpoint, the regenerative effect of the S&S Hernia System aligns with the goal of the treatment: to halt degeneration and promote the regeneration of the compromised components of the herniated abdominal wall. To validate the regenerative capabilities of the S&S device positioned within the muscular environment of the abdominal wall, it is crucial to emphasize the ingrowth of myocytes—key components of the abdominal barrier—into the 3D scaffold of the device. Therefore, the primary objective of this study was to confirm the existence and amount of these specialized connective structures within the device. A secondary goal was to document the progression of muscle ingrowth by time.

The results of this study confirmed that, even in the short term post-implantation, the development of connective tissue in the device was composed of viable tissue with several spots of emerging muscle elements in early developmental stages. In this phase, the nuclei of the myocytes were already formed, though the striated muscle structure was not yet fully developed. The muscle elements exhibited moderate basophilia with vesiculated nuclei and prominent nucleoli.

As the study progressed to the mid-term phase (approximately 3–4 months), there was a notable increase in both the quantity and quality of muscle elements within the device. These elements progressively evolved, eventually displaying characteristics of mature muscle fibers: spindle-shaped myocytes with hyperchromic small nuclei and eosinophilic cytoplasm.

In the long-term phase (5–8 months post-implantation), the structure of the myocytes closely resembled that of regular human muscle tissue, with widespread muscle ingrowth observed within the implant fibers. Overall, the histological analysis of the specimens excised in the long term revealed that the evidenced myocytes exhibited the typical structure of organized muscle tissue, comparable to normal human muscle bundles [32,33,34,35,36].

The presence and progressive increase in NGF-positive cells within the TPE matrix of the S&S scaffold across all time points strongly suggest that neurotrophic signaling may play a pivotal role in orchestrating the myogenic regenerative response. In the early postoperative phase, NGF expression coincided with the emergence of embryonic myocytes within a hydrated connective tissue matrix, indicating the initiation of muscle cell differentiation. By the mid- and long-term stages, this expression pattern evolved in parallel with the maturation of muscle fibers and the spatial organization of muscle bundles, reinforcing the hypothesis that NGF may contribute to both the proliferation and differentiation of myogenic precursors. These findings are in line with prior studies that implicate NGF in skeletal muscle repair and support the concept that the dynamic environment within the S&S scaffold provides favorable biomechanical and biochemical conditions to stimulate sustained neomyogenesis [37,38,39]. The correlation between scaffold architecture, dynamic compliance, and growth factor expression warrants further investigation, particularly regarding the potential regulatory role of neurotrophins in hernia repair biology.

These distinctive histological and immunohistochemical outcomes were further validated by statistical analysis conducted across all stages of the investigation, reinforcing the concept of a regenerative biological response induced by the dynamic behavior of the S&S Hernia System.

It is worth noting that the regenerative biological response observed in this newly designed hernia device is not entirely unprecedented, as similar features have been reported with another dynamic regenerative scaffold developed for inguinal hernia repair [13,18]. This device, known as ProFlor, represents the first generation of regenerative scaffolds specifically engineered for inguinal hernia treatment. Unlike the S&S device, ProFlor requires conventional surgical dissection of the abdominal wall—an approach that, due to surgical trauma, is commonly associated with tissue swelling, postoperative pain, and prolonged recovery. In contrast, the S&S device marks a significant advancement, as it can be positioned in a matter of seconds without any dissection, incision, or tissue trauma.

Despite these procedural differences, both devices exhibit a comparable regenerative biological response, as confirmed by histological analysis. This regenerative activity—encompassing all components of the abdominal wall—has been scientifically validated through a series of publications, which attribute the observed tissue regeneration to the dynamic behavior of the ProFlor scaffold. Taken together, these findings suggest the emergence of a new category in hernia repair technology: dynamic regenerative scaffolds.

Nevertheless, this investigation has a notable limitation: it does not fully explain the mechanisms underlying the observed neomyogenesis. Preliminary hypotheses suggest that specific growth factors may play a role in stimulating the development of new myocytes and muscle bundles. Ongoing research is focused on clarifying these potential mechanisms.

## 5. Conclusions

This study introduces and validates the regenerative potential of the Stenting and Shielding (S&S) Hernia System—a newly designed, dissection-free, dynamic implant for abdominal hernia repair. Unlike conventional flat meshes that trigger a passive, fibrotic response and may contribute to complications such as chronic pain and implant shrinkage, the S&S device demonstrated a biologically active, regenerative response characterized by the ingrowth of vascular, neural, and muscle tissue within its 3D scaffold. Histological and statistical analyses across short-, mid-, and long-term time points confirmed the progressive development of newly formed muscle structures, culminating in organized, mature muscle bundles that closely resemble native tissue. These findings suggest that dynamic responsiveness to physiological movement is a key determinant of regenerative outcomes in hernia repair. Compared to its predecessor, the ProFlor device, the S&S Hernia System achieves similar regenerative efficacy while introducing a major surgical innovation: laparoscopic implantation without dissection, trauma, or fixation. This feature not only simplifies the surgical procedure but also minimizes patient morbidity, positioning the device as a significant advancement in hernia surgery. Although the precise molecular mechanisms behind the observed neomyogenesis remain to be fully elucidated, the evidence supports a new paradigm in hernia repair—one that favors functional regeneration over mechanical reinforcement. The concept of dynamic regenerative scaffolding is thereby reinforced and expanded by the findings of this investigation. Future studies will be necessary to investigate the molecular signaling involved in the regenerative process and to confirm the clinical applicability of the S&S device in human models. Nevertheless, the present results lay the foundation for a transformative approach to abdominal wall reconstruction, aligning surgical technique with biologically coherent, patient-centered outcomes.

## Figures and Tables

**Table 1:** Technical specifications of the Thermoplastic Elastomer (TPE) material utilized for the injection molding of the Stenting and Shielding Hernia System.

TPE Technical Specifications	Value	Unit	Test Standard	Reference
ISO Data				
Tensile Strength	16	MPa	ISO 37	[20]
Strain at break	650	%	ISO 37	[20]
Compression set at 70 °C, 24 h	54	%	ISO 815	[21]
Compression set at 100 °C, 24 h	69	%	ISO 815	[21]
Tear strength	46	kN/m	ISO 34-1	[22]
Shore A hardness	89	-	ISO 7619-1	[23]
Density	890	kg/m^3^	ISO 1183	[24]

## Data Availability

All data supporting the reported results are available upon request from the corresponding author. The data are not publicly available due to intellectual property and patent-related restrictions, as the device is under active development and patent protection.

## Supplementary Materials

The following supporting information can be downloaded at https://www.mdpi.com/article/10.3390/bioengineering12080883/s1, Figure S1. Representative immunohistochemistry images showing NGF staining (antibody Abcam ab52918) in excised porcine specimens. (A) Fascicles of skeletal muscle fibers displaying specific NGF positivity. NGF 100X. (B) A well-constituted nervous structure close to a vein and an artery within the same tissue sample exhibiting selective NGF staining. NGF 200X. These images demonstrate the antibody’s specificity for both muscular and neural elements in porcine tissues, supporting its suitability for the present study.
