# Supplementary material for: Dynamic Behavior of the Stenting & Shielding Hernia System Fosters Neomyogenesis in Experimental Porcine Model
Source: Bioengineering (Basel). 2025 Aug 19;12(8):883. doi: 10.3390/bioengineering12080883 (PMC12383935; doi:10.3390/bioengineering12080883)
Supplement: Supplementary file 1 [file bioengineering-12-00883-s001.zip › bioengineering-3687394-SM.pdf]

bioengineering

MDPI

Article

Dynamic Behavior of the Stenting & Shielding Hernia System Fosters Neomyogenesis in Experimental Porcine Model

Giuseppe Amato [1], Roberto Puleio [2], Antonino Agrusa [1], Vito Rodolico [3], Luca Cicero [4], Giovanni Cassata [4], Giuseppe Di Buono [1], Emanuele Battaglia [1], Claudia Neto [5], Giorgio Romano [5], William Ra [5] and Giorgio Romano [1]

[1] Department of Precision Medicine in Medical, Surgical and Critical Areas, University of Palermo, 90127 Palermo, Italy; antonino.agrusa@unipa.it (A.A.); giuseppe.dibuono@unipa.it (G.D.B.); emanuele.battaglia@policlinico.pa.it (E.B.); giorgio.romano@unipa.it (G.R.)
[2] Department of Pathologic Anatomy and Histology, Istituto Zooprofilattico Sperimentale della Sicilia (IZSS Palermo), 90129 Palermo, Italy; roberto.puleio@izssicilia.it
[3] Department PROMISE, Section Pathological Anatomy, University of Palermo, 90127 Palermo, Italy; vito.rodolico@unipa.it
[4] CEMERIT—Experimental Zooprophylactic Institute of Sicily Palermo, 90129 Palermo, Italy; luca.cicero@izssicilia.it (L.C.); giovanni.cassata@izssicilia.it (G.C.)
[5] Postgraduate School of General Surgery, University of Palermo, 90127 Palermo, Italy; neto.claudia98@gmail.com (C.N.); giorgioromano95@gmail.com (G.R.); williamra95@gmail.com (W.R.)
* Correspondence: amatomed@gmail.com; Tel.: +39337963197; Fax: +390916552836

Supplemental material:

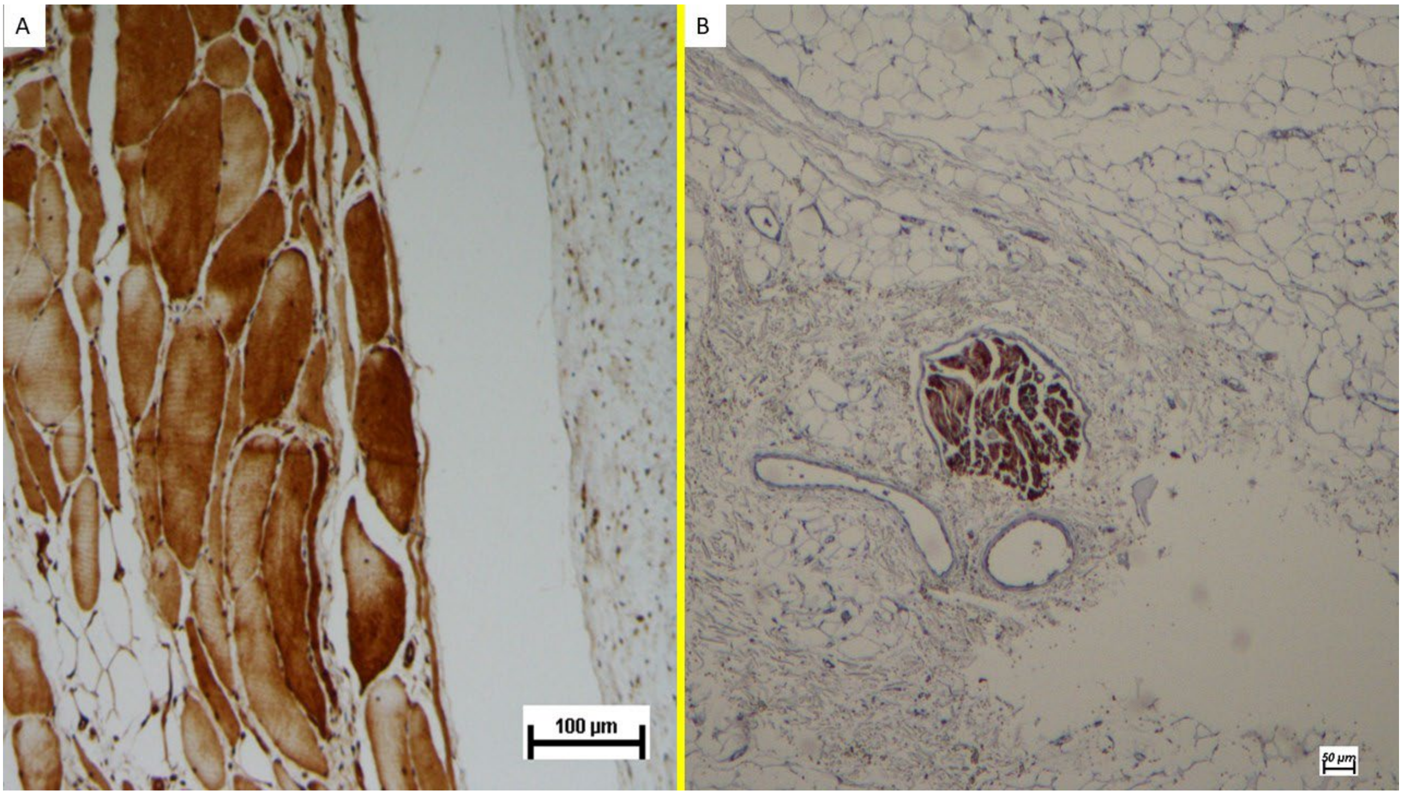

Figure S1. Representative immunohistochemistry images showing NGF staining (antibody Abcam ab52918) in excised porcine specimens. (A) A fascicles of skeletal muscle fibers displaying specific NGF positivity. NGF 100X – B: A well constituted nervous structure close to a vein and an artery within the same tissue sample exhibiting selective NGF staining. NGF 200X. These images demonstrate the antibody's specificity for both muscular and neural elements in porcine tissues, supporting its suitability for the present study.

 https://doi.org/10.3390/bioengineering12080883
